# Mixed and central venous oxygen saturation are not interchangeable in patients with cardiogenic shock after cardiac surgery

**DOI:** 10.1186/cc12118

**Published:** 2013-03-19

**Authors:** S Romagnoli, P Balsorano, F Landucci, A De Gaudio

**Affiliations:** 1 AOUC Careggi, Florence, Italy

## Introduction

Mixed venous oxygen saturation (SVO_2_) represents a well-recognized parameter of oxygen delivery (DO_2_)-consumption (VO_2_) mismatch and its use has been advocated in critically ill patients in order to guide hemodynamic resuscitation [1] and oxygen delivery optimization. Nevertheless, the pulmonary artery catheter (PAC) is not readily available and its use is not devoid of risks. Furthermore, its use has been decreasing in recent years in surgical and cardiac surgical patients as the benefit of guiding therapy with this device is unclear [2-4]. Central venous oxygen saturation (ScVO_2_) has been suggested as an alternative to SVO_2 _monitoring due to its feasibility in several settings. Unfortunately concerns arise from its capability to correlate with SVO_2_, the relationship being influenced by several factors, such as hemodynamic impairment and pathological process. Hemodynamic instability and shock often complicate cardiac surgery, and the SVO_2_-ScVO_2 _relationship has not been specifically investigated in this setting. The aim of this study is to compare SVO_2 _and ScVO_2 _values in patients with cardiogenic shock after cardiac surgery.

## Methods

A prospective observational study was designed and conducted. Inclusion criteria were: patients who had underwent elective or urgent/emergent cardiac surgery, with cardiac index (CI) <2.5 l/minute/m^2 ^estimated by means of a PAC, left ventricle ejection fraction (LVEF) <40%, lactate >2 mmol/l, age >18 years. A central venous catheter (CVC) and a PAC were inserted for each patient before surgery in the same right internal jugular vein in accordance with standard procedure. Proper position of the PAC was confirmed with pressure tracings and chest X-ray. Mixed and central venous blood samples were collected from the distal ports of the PAC and CVC respectively 30 minutes after ICU admission, and every 6 hours for a total of three samples in a 24-hour period for each patient. All blood samples were analyzed by a co-oximeter (Radiometer ABL800 flex; Radiometer, Copenhagen, Denmark). Statistical analysis was performed by Stats Direct (Ver.2.5.8, Cheshire, UK) and GraphPad (Vers. Prism 4.0; San Diego, CA, USA). All data were tested for normal distribution with the Kolmogorov-Smirnov test. Statistical analysis was performed by linear regression analysis. The agreement between absolute values of ScvO_2 _and SvO_2 _were assessed by the mean bias and 95% limits of agreement (LOA) ((mean bias ± 1.96)×standard deviation) according to the method described by Bland and Altman [5].

## Results

**Figure 1:**
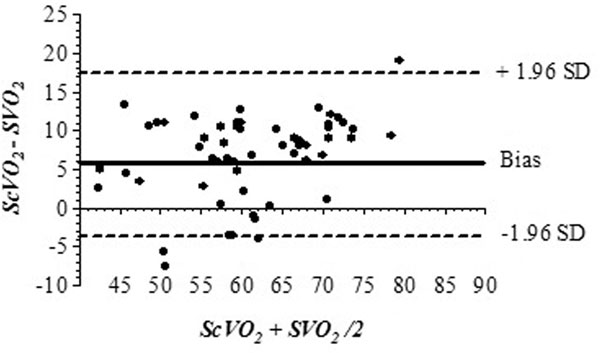


A total of 20 patients were enrolled. In 18 out of 20 cases all three blood samples were collected. In two patients only two blood samples were drawn as they exited the inclusion criteria. Linear regression analysis between the two variables resulted in an *r*^2 ^of 0.708. Bland-Altman analysis (Figure 1) for the pooled measurements of SvO_2 _and ScvO_2 _showed a mean bias and LOA of 6.82% (SD of bias 5.3) and -3.71 to +17.3% respectively.

## Conclusion

ScVO_2 _has been advocated as an attractive and simple indicator of DO_2_-VO_2 _mismatch [6]. Its role as a surrogate of the wellestablished SVO_2 _has been investigated in several settings, and it has been purposed in the hemodynamic resuscitation of critically ill septic patients [1]. Nevertheless, the SVO_2_-ScVO_2 _relationship can be influenced by several factors due to ScVO_2 _dependency from global blood flow redistribution that can occur during hemodynamic impairments. It has been shown previously that in healthy people ScVO_2 _values tend to underestimate SVO_2 _values, due to the higher oxygen content from inferior vena cava [7]. During circulatory shock, not homogeneous oxygen extraction and regional blood flow redistribution make SVO_2 _a more reliable parameter suggesting the global adequacy of cardiac output rather than ScVO_2_. In this study we aimed at evaluating SvO_2_-ScVO_2 _differences in patients with cardiogenic shock, as defined by hyperlactatemia, low CI, and LVEF <40%, after cardiac surgery. Our results highlighted a great variability for these two parameters, with a clinically unacceptable mean bias and LOA. As expected, ScVO_2 _values were consistently higher.
